# ELEPHANTIASIS OF THE EXTERNAL GENITALIA: A SEQUEL TO CUTANEOUS TUBERCULOSIS

**DOI:** 10.4103/0019-5154.48988

**Published:** 2009

**Authors:** L Padmavathy, L Lakshmana Rao, K Chockalingam, N Ethirajan, M Dhanlakshmi

**Affiliations:** *From the Dermatologist, Urban Health Center, Division of Community Medicine, Annamalai University, Tamil Nadu, India*; 1 *From the Department of Patholgy, Annamalai University, Tamil Nadu, India*; 2 *From the Department of Dermatology, Venereology, and Leprosy, Annamalai University, Tamil Nadu, India*; 3 *From the Department of Community Medicine, Rajah Muthiah Medical College, Annamalai University, Tamil Nadu, India*

**Keywords:** *Cutaneous tuberculosis*, *diabetes mellitus*, *elephantiasis*, *gumma*, *lupus vulgaris*, *scrofuloderma*

## Abstract

Tuberculosis continues to be an important public health problem and cutaneous tuberculosis constitutes a minor proportion of extra pulmonary manifestations of tuberculosis. Elephantiasis of the external genitalia, as a sequel to cutaneous tuberculosis, in a 40-year-old diabetic lady is being reported for its rarity. The patient also had lesions of healed scrofuloderma of 27 years’ duration, in both axillae, with residual pedunculated nodules.

## Introduction

With the advent of HIV infection, there is a resurgence of tuberculosis in both developing and developed countries. Cutaneous tuberculosis constitutes a minor proportion of the extra-pulmonary manifestations of tuberculosis.

Elephantiasis is the dramatic end result of a variety of obstructive diseases of the lymphatic system, commonly affecting the arms, legs and genitalia.[1] Genital elephantiasis is a common sequel to filariasis and lymphogranuloma venereum. Rarely can it result from irradiation of the inguinal lymph nodes or Donavanosis, carcinomas and tuberculosis.[2] Lupus vulgaris is a progressive form of tuberculosis, occurring in people with moderate or high degree of immunity, and is more common in women.[3] Gumma is a term for metastatic tuberculous abscesses,[4] where caseating tuberculosis develops in the cutis or subcutis, independent of the underlying lymph nodes, and gradually invades the subjacent skin and eventually ulcerates.[5]

A case of cutaneous tuberculosis with varied morphology - healed scrofuloderma in both the axillae, lesions suggestive of lupus vulgaris on the thighs and lesions of gumma in the gluteal region and elephantiasis of the external genitalia is being reported for its rarity.

## Case Report

A 40-year-old female patient presented with the complaint of swelling in the external genitalia for one year and leucorrhea of 20 days’ duration. There was a history of fever, but no cough. She was a known diabetic, on treatment, with inadequate glycemic control. She had swelling and discharging nodules in both axillae, from 13 years of age (27 years’ duration), and was on irregular antituberculous treatment. Her father had pulmonary tuberculosis.

**Figure 1:**
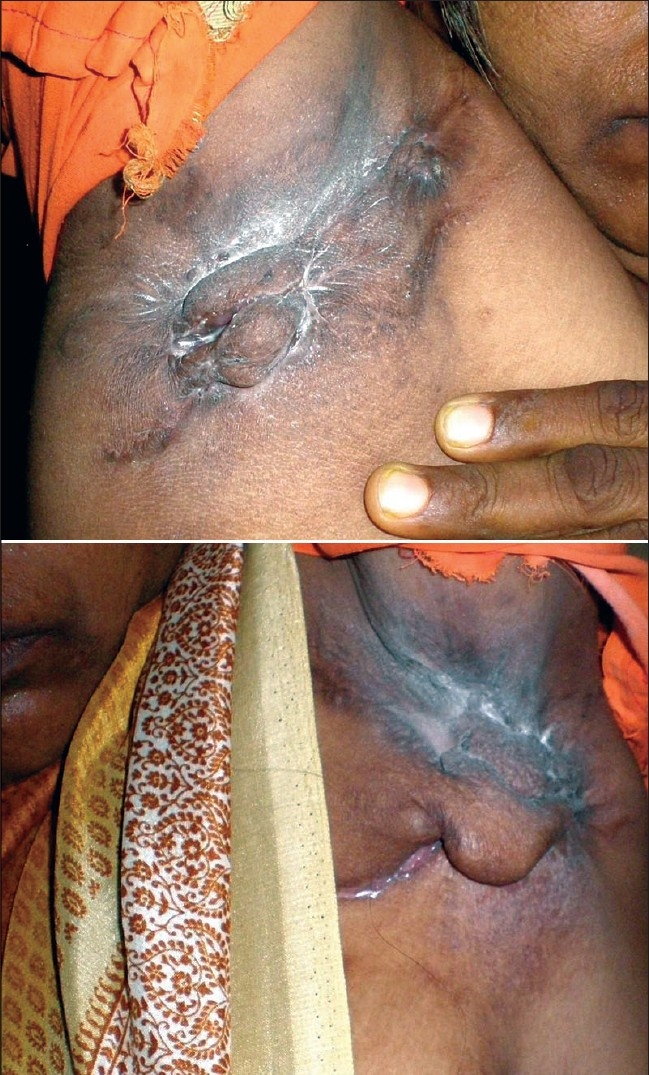
Multiple scars (bridge scars) in both axillae with matted and non tender lymph nodes and pedunculated, firm nodules associated with intertrigo

Cutaneous examination revealed multiple scars (bridge scars) in both axillae [Figure 1] with matted and non tender lymph nodes and pedunculated, 4cm × 2cm, firm nodules, associated with intertrigo in both axillae. There were no active discharging sinuses in the axillae. There was candidal intertrigo between the skin folds in the nodules [Figure 1].

**Figure 2:**
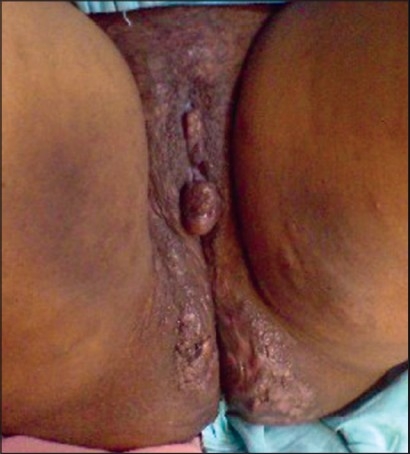
Swelling of the external genitalia, with pedunculated polypoid growth 3 cm × 3 cm, extending from the left labium minus

**Figure 3:**
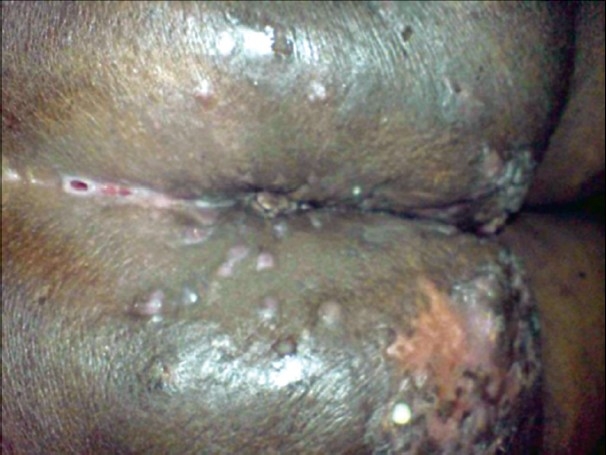
Indurated, firm plaques, with multiple discharging sinuses over the gluteal region, in a bilaterally symmetrical distribution

**Figure 4:**
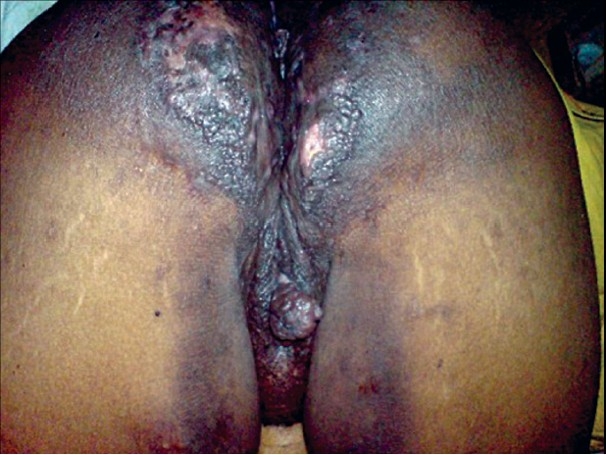
Indurated, firm plaques, with multiple discharging sinuses over the perineum

External genitalia showed swelling, with a pedunculated, polypoid growth, 3cm × 3cm, extending from the right labium minus [Figure 2]. There were multiple skin colored papules attached to both labia minora. There was thick curdy white discharge per vaginum. Verrucous hyperkeratotic plaques with papillomatosis and scarring were seen on the medial aspect of both thighs. Indurated, firm plaques, 23cm × 10cm, with multiple discharging sinuses, were seen on the perineum and extending onto the gluteal region, in a bilaterally symmetrical distribution [Figure 3, Figure 4]. Per vaginal examination did not reveal any abnormality, either in the uterus or the cervix. Inguinal and femoral lymphadenopathy could not be made out due to the gross thickening of the skin in the region. Other systems were clinically normal.

Except for anemia (Hb at 8.6gm/dl) and an elevated ESR of 110mm/1^st^ hour, other hematological and biochemical parameters were within normal limits. Total serum proteins were 8.3gm%; albumen 4.3gm%; and globulin 4gm%. The patient's blood sugar was initially 220mg/dl, but was brought under control by increasing the dose of oral hypoglycemic agents.

Roentgenogram chest and Ultrasonography (USG) of the abdomen, including the pelvic organs, revealed no abnormality. Sputum was negative for acid fast bacilli (AFB). The patient was HIV negative and her VDRL test was non reactive, but the Mantoux test was positive.

**Figure 5:**
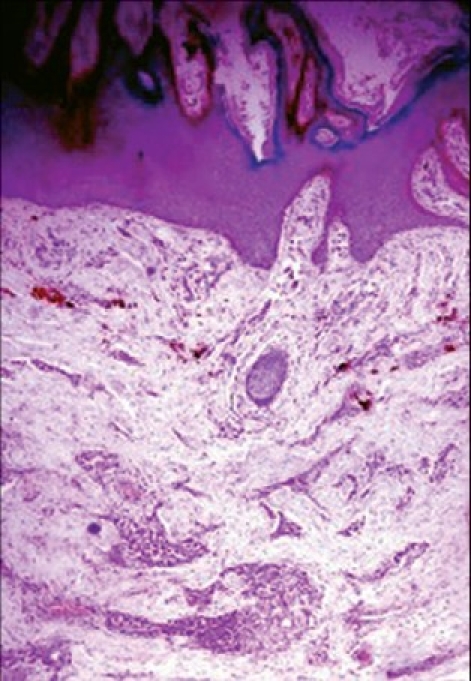
Skin biopsy showing hyperkeratosis, papillomatosis, acanthosis of epidermis and epithelioid cell granulomas in dermis (H and E) × 20

**Figure 6:**
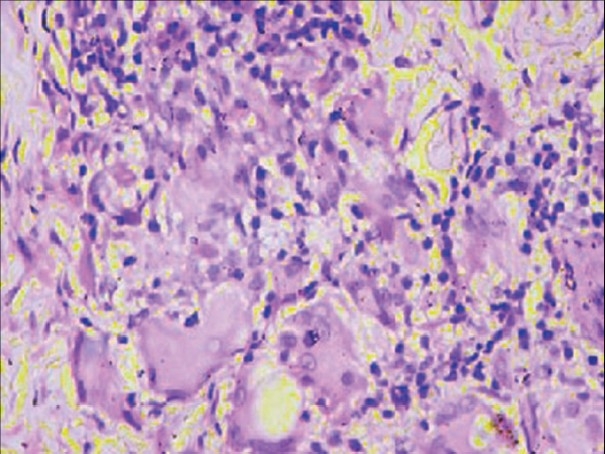
Epithelioid cell granulomas with Langhan's giant cells (H and E, ×40)

A clinical diagnosis of scrofuloderma in both axillae and genital elephantiasis as a sequel to cutaneous tuberculosis was entertained. The possibility of tuberculous gumma was also considered, in view of the discharging sinuses in the perineum. However, skin biopsy from the verrucous plaque on the medial aspect of thigh showed marked hyperkeratosis and papillomatosis of the epidermis; epithelioid cell granulomas with inflammatory infiltrate composed of neutrophils and lymphocytes in the dermis [Figure 5, Figure 6]. *Staphylococcus aureus* was found in smears from the discharge, while both tissue biopsy specimens as well as smears from the discharge were negative for AFB by Z-N stain.

Besides, the patient was Mantoux positive and gumma is more common in patients with poor immunity and Mantoux negativity. A second biopsy from the sinuses in the gluteal region could not be undertaken due to non compliance by the patient.

The secondary bacterial infection was brought under control with suitable antibacterial antibiotics. The patient was started on standard anti tuberculous therapy (ATT), category-I, with Isoniazide 600mg, Rifampicin 450mg, Pyrazinamide 1500mg and Ethambutol 1200mg, and Pyridoxine (B6) 10mg, thrice weekly. In a few weeks, the sinuses began to dry up gradually; the perianal lesions and the plaques on the medial aspect of the thighs flattened and showed signs of healing.

## Discussion

An ulceration that tore into the flesh like the ravages of a wolf probably fitted the clinical description and explains the word ‘lupus’ which means wolf. The commonness of this condition in earlier times accounts for the adjective ‘vulgaris’ in lupus vulgaris.[6] The earliest description of lupus vulgaris was by Erasmus Wilson, in 1865. The other synonyms for this condition are: tuberculosis luposa and tuberculosis luposa cutis.[7]

Tuberculous gumma results from hematogenous dissemination from a primary site of tuberculosis, especially in malnourished children with impaired immunity.[8] Tuberculous gumma differs from scrofuloderma, in arising independently, of an underlying lymph node.[5] In our patient, the sinuses were present in the gluteal region. Our patient had a positive Mantoux test, indicating moderate immunity.

The clinical variants of lupus vulgaris are many and include hypertrophic, ulcerative, vegetative, papular, nodular and mucosal lesions. Hypertrophic forms may present as tumorous growths of soft consistency or may show epithelial hyperplasia with the production of hyperkeratotic masses, as was observed in our patient. Edema, lymphatic stasis and recurrent erysipelas, elephantiasic thickening and vascular dilatation accompany these tumor like forms and may cause gross deformity,[9] leading to a clinical picture, as in our patient, on the medial aspect of the thighs.

Lupus vulgaris (LV) originates from a tuberculous condition or a clinically inapparent focus elsewhere in the body, by hematogenous, lymphatic or contiguous spread.[9] In our patient the axillary lymphadenopathy, which started when she was 13 years of age, could be presumed to be the primary focus, as her X-ray of the chest was normal. In about 30% of the cases, LV could be preceded by scrofuloderma,[9] as was observed in our patient too, though the LV lesions were far removed from the scrofuloderma lesions. Uncontrolled diabetes might have contributed to the hematogenous dissemination of bacilli.

The face is the most common site of the involvement of LV among patients from the Western countries.[9] In India, the lower extremities, especially the buttocks, seem to be affected more commonly, as noted in our patient. The probable hypothesis is that the bacilli lying dormant for years are reactivated by trauma and non-specific inflammation.[3]

The histological changes in the epidermis depend on the morphology of the lesion - either atrophy or hyperplasia is prominent.[10] In our patient, there was marked hyperkeratosis, papillomatosis and acanthosis, corresponding to the clinical picture on the medial aspect of the thigh. The patient refused a second biopsy from the discharging sinuses on the gluteal region, which probably could have been helpful in establishing a definitive diagnosis of gumma. However, it would have been an academic exercise, since the tuberculous pathology was revealed by a previous biopsy from an adjacent site.

Elephantiasis is applied to many dermatological conditions that ultimately result in severe lymphatic obstruction and stasis. The skin becomes discolored and patches of warty growths occur in the affected area,[7] as was observed in our patient.

Elephantiasis of the vulva due to extensive destruction of the lymph nodes in the inguinal and femoral region by tuberculous process, though uncommon, was reported.[1,11] However, in our patient, due to the gross thickening and verrucosity of the lesions on the medial aspect of thigh, the inguinal and femoral lymph nodes could not be palpated.

In view of the varied morphology of the lesions, with hyperkeratotic verrucous plaques on the medial aspect of thighs and the discharging sinuses on the perineum and gluteal region, in addition to the long standing healed lesions of scrofuloderma in the axillae, this case could not be classified exactly and, hence, was labeled as cutaneous tuberculosis, leading to elephantiasis of the external genitalia.

With the initiation of anti tuberculous treatment, the perineal lesions began to regress. The discharging sinuses healed. The pedunculated polypoid growth from the labium minus was excised. There was no significant alteration in the swelling of the axillae, for which she was referred to a surgeon. Her candidal vulvovaginitis was treated with topical and systemic antifungal medications.

The present case is being highlighted for the rarity of elephantiasis of the genitalia, as a sequel to cutaneous tuberculosis.
